# Radiomic analysis for predicting prognosis of colorectal cancer from preoperative ^18^F-FDG PET/CT

**DOI:** 10.1186/s12967-022-03262-5

**Published:** 2022-02-02

**Authors:** Lilang Lv, Bowen Xin, Yichao Hao, Ziyi Yang, Junyan Xu, Lisheng Wang, Xiuying Wang, Shaoli Song, Xiaomao Guo

**Affiliations:** 1 Department of Radiotherapy, Fudan University Shanghai Cancer Center, No.270 Dong’an Road, Xuhui district, Shanghai, 200032 China; 2 School of Computer Science, The University of Sydney, Sydney, NSW Australia; 3 Department of Nuclear Medicine, Fudan University Shanghai Cancer Center, No.270 Dong’an Road, Xuhui district, Shanghai, 200032 China; 4 Department of Automation, Shanghai Jiao Tong University, Shanghai, 200240 China; 5 Department of Oncology, Shanghai Medical College, Fudan University, Shanghai, China; 6 Center for Biomedical Imaging, Fudan University, Shanghai, China; 7 Shanghai Engineering Research Center of Molecular Imaging Probes, Shanghai, China

**Keywords:** Stage III Colorectal cancer, ^18^F-FDG PET/CT, Radiomics, Prognosis, Prediction

## Abstract

**Background:**

To develop and validate a survival model with clinico-biological features and ^18^F- FDG PET/CT radiomic features via machine learning, and for predicting the prognosis from the primary tumor of colorectal cancer.

**Methods:**

A total of 196 pathologically confirmed patients with colorectal cancer (stage I to stage IV) were included. Preoperative clinical factors, serum tumor markers, and PET/CT radiomic features were included for the recurrence-free survival analysis. For the modeling and validation, patients were randomly divided into the training (n = 137) and validation (n = 59) set, while the 78 stage III patients [training (n = 55), and validation (n = 23)] was divided for the further experiment. After selecting features by the log-rank test and variable-hunting methods, random survival forest (RSF) models were built on the training set to analyze the prognostic value of selected features. The performance of models was measured by C-index and was tested on the validation set with bootstrapping. Feature importance and the Pearson correlation were also analyzed.

**Results:**

Radiomics signature (containing four PET/CT features and four clinical factors) achieved the best result for prognostic prediction of 196 patients (C-index 0.780, 95% CI 0.634–0.877). Moreover, four features (including two clinical features and two radiomics features) were selected for prognostic prediction of the 78 stage III patients (C-index was 0.820, 95% CI 0.676–0.900). K–M curves of both models significantly stratified low-risk and high-risk groups (*P* < 0.0001). Pearson correlation analysis demonstrated that selected radiomics features were correlated with tumor metabolic factors, such as SUVmean, SUVmax.

**Conclusion:**

This study presents integrated clinico-biological-radiological models that can accurately predict the prognosis in colorectal cancer using the preoperative ^18^F-FDG PET/CT radiomics in colorectal cancer. It is of potential value in assisting the management and decision making for precision treatment in colorectal cancer.

*Trial registration* The retrospectively registered study was approved by the Ethics Committee of Fudan University Shanghai Cancer Center (No. 1909207-14-1910) and the data were analyzed anonymously.

**Supplementary Information:**

The online version contains supplementary material available at 10.1186/s12967-022-03262-5.

## Introduction

Colorectal cancer (CRC) is one of the most commonly diagnosed cancers all over the world, though its epidemiology is different in various regions [1]. It is also one of the leading causes of cancer-related mortality despite the advancement in treatment strategies [2, 3]. The prognosis of CRC is one of the essential factors in patient management and selection of treatment strategies [4]. Tumor Node Metastasis (TNM) staging classification system plays an important role in colorectal cancer prognostication [4–6]. But TNM staging system cannot accurately differentiate the prognosis of colon cancer with stage II and III. A series of disease characteristics known to affect the survival of colorectal cancer were not directly included in the TNM staging system, such as age, gender, location of primary disease, tumor grade, lymphatic vessel and peripheral nerve infiltration, intestinal obstruction or perforation and BRAF and KRAS Mutations [7–10]. Blood and stool protein markers have also been investigated to identify patients with favorable and poor prognosis [11, 12]. Several studies were dedicated to other prognostic factors in patients with MSI status and chromosome 18q loss of heterozygosity in the coding place [13, 14]. Several studies have attempted to provide clinical assistance in the management strategies of colorectal cancer by utilizing important imaging prognostic features, such as depth of tumor spread, presence of malignant lymph nodes, tumor deposits, extramural vascular invasion, and differentiation of mucinous from nonmucinous tumors [15].

For staging primary colon cancer, contrast-enhanced computed tomography (CT) scans achieved accuracies ranging from 60 to 80% [16–19]. MRI features are useful in diagnosing locally advanced rectal tumors and also are helpful to assess regional nodal involvement and treatment response [20, 21]. However, the above anatomical imaging is based on morphology, cannot provide the metabolic characteristics of the tumor lesion. ^18^F-fluoro-2-deoxy-d-glucose Positron emission tomography/computed tomography (^18^F-FDG PET/CT) can sensitively provide the molecular and functional information of not only the primary tumor, but also distant metastasis lesion and the recurrent disease by one-time imaging [22]. ^18^F-FDG, like glucose, is transported into cells by glucose transporters, where it is transformed into ^18^F-FDG-6-phosphate (^18^F-FDG-6-P). Because ^18^F-FDG-6-P cannot be metabolized further, it becomes trapped inside cells. Compared with normal tissue, tumor cells are highly proliferative and have a high glucose metabolic rate. Therefore, tumor cells accumulate more ^18^F-FDG-6-P than normal cells [23]. The degree of metabolic activities or tissue uptake of FDG was expressed as a standardized uptake value (SUV). In colorectal cancer, cells showed increased glycolysis metabolism. PET can be quantitatively evaluated and compared in studies (e.g., pre- and post-treatment)[24] using SUV, thus assisting to obtain insights into tumor biology. The correlation between FDG metabolism in PET and tumor proliferation rates that may in turn help determine the prognosis of CRC. It is known that the scan highly depends on the patient's physical condition due to a 6-h fast.

Radiomics is a promising translational research field that can provide quantified tumor heterogeneity information from medical images in a non-invasive manner. Studies have shown that radiomic features based on CT or MRI were related to the prognosis of colorectal cancers [25–27]. PET/CT radiomics has achieved success in wide range of cancers [28–31]. For example, PET/CT radiomics has successfully predicted the prognosis of various malignancies including gastric cancer [32], nasopharyngeal carcinoma [33]. lung cancer, breast cancer and other tumors [34–36]. There have been several studies of PET/CT radiomics in rectal cancer, however, not in colon cancer [37–40]. Other PET/CT radiomics studies focused on colorectal cancer patients with metastasis [41, 42]. In addition, studies used texture analysis instead of complete radiomics to study the prognosis of colorectal cancer with a relatively small number of cases[37, 43]. Some studies reported the metabolic phenotype could predict genetic alterations of colorectal cancer by ^18^F-FDG PET/CT radiomics [44]. Therefore, we conclude that the prognostic value of PET/CT radiomics based on primary colorectal cancer has not been deeply explored, especially in stage III patients who accounts for a high proportion of colorectal cancer patients. In this study, we investigated the prognostic value of ^18^F-FDG PET/CT-based radiomics features using machine learning for CRC patients of all stages and then applied the same method on CRC patients with stage III to analyze the differences.

## Materials and methods

### Patients collection

The study was approved by Ethics Committee of Fudan University Shanghai Cancer Centre and Institutional Review Board for clinical investigation. In our study, 196 patients diagnosed with colorectal cancer between January 2010 and July 2018 were retrospectively collected from an electronic database in Fudan University Shanghai Cancer Centre. Patients were followed up until July 2020. All patients who met the following criteria were enrolled: (1) Patients received surgery at the primary colorectal lesion and the final pathology was colorectal adenocarcinoma or mucinous adenocarcinoma; (2) Immunohistochemical results also was received; (3) Patients received no preoperative treatment and underwent preoperative ^18^F-FDG PET/CT. Thus, the difference in tumor metabolism after adjuvant therapy was avoided; (4) Patients did not receive any chemotherapy, radiation therapy, or molecular targeted therapy before ^18^F-FDG PET/CT scans yet; (5) Patients were not lost to follow-up. We reviewed 243 patients diagnosed with colorectal cancer in total and finally enrolled 196 patients for this study.

### ^18^F-FDG PET/CT protocol and imaging interpretation

^18^F-FDG PET/CT scans were performed using a PET/CT scanner (Siemens Medical Systems, Biograph 16 HR). All patients fasted for at least 6 h before ^18^F-FDG administration and glucose levels in the peripheral blood were confirmed to be 10 mmol/L or less before the ^18^F-FDG injection (7.4 MBq/kg (0.2 mCi/kg) of body weight) in this study. The scanning included the area from the upper thigh to the skull. Data acquisition started approximately 1 h after the injection. The low-dose CT scans were obtained with the following parameters: 40–60 mA, 120 kV, 0.6-s tube rotation, and 3.75-mm section thickness. The spatial resolution of PET images was 168 × 168 × 172 with voxel size 4.06 × 4.06 × 5 ${mm}^{3}$, while the resolution of CT images was 512 × 512 × 172 with voxel size 1.37 × 1.37 × 5 ${mm}^{3}$. For quantitative analysis, ^18^F-FDG accumulation on a workstation was assessed by two experienced nuclear medicine physicians through calculating the standardized uptake value (SUV), metabolic tumor volume (MTV) and total lesion glycolysis (TLG) in the regions of interest placed over the suspected lesions and the normal liver. SUV was calculated in a pixel as (radioactivity) / (injected dose/body weight). TLG was calculated as (mean SUV) × (MTV), in which MTV was measured with setting a margin threshold as SUV of 2.5. All values of SUVmax, MTV, and mean SUV were automatically measured by analysis software for each lesion. For evaluating metastatic CRC, the highest SUV in a metastatic tumor was taken as SUVmax and the mean SUV was taken as SUVmean.

### Medical image delineation

The Volume of Interest (VOIs) in the tumor was segmented slice by slice by two attending nuclear medicine physicians respectively. The open-source software ITK-Snap [45] was used for segmentation. If the two opinions were different, they discussed and made the final decision together. The physicians segmented tumors only on the basis of imaging findings and did not consider pathological findings. Since the PET/CT images were co-registered, only the VOIs of PET images were manually segmented, and then resampled to CT images through coordinate transformation and interpolation. The resulting VOIs for CT images were validated by a radiologist.

### Radiomics feature extraction

**Fig. 1:**
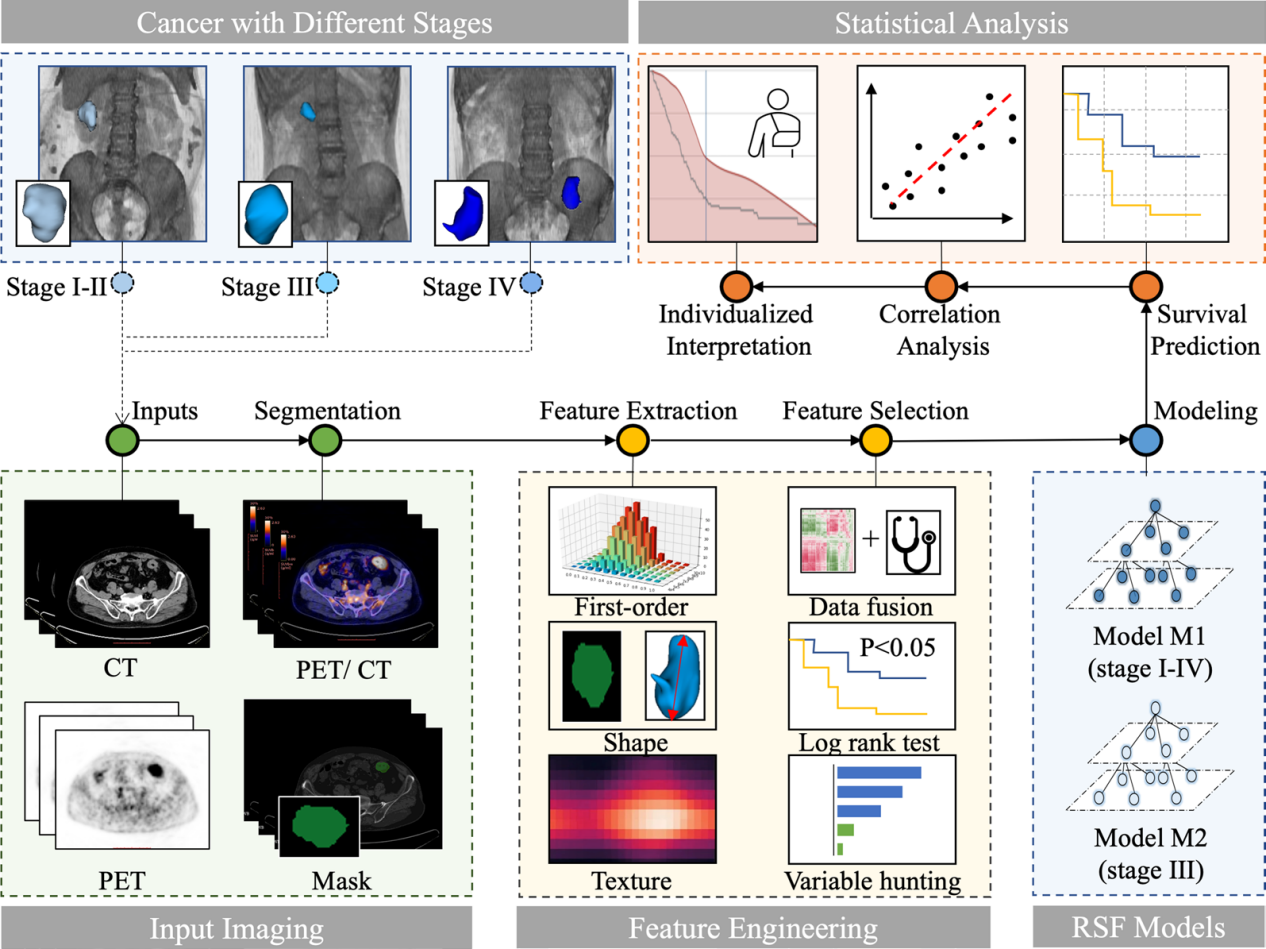
Radiomics workflow. Input PET/CT was collected from patients with colorectal cancer (Stage I–IV). Feature engineering was used to extract radiomics features from region of interests in PET/CT images and to select important features via log-rank test and variable hunting. Two random survival forest (RSF) models M1 and M2 were constructed followed by statistical analysis including survival prediction, correlation analysis and individualized interpretation

Radiomics Workflow was illustrated in Fig. 1 including three main modules: Feature Engineering, Random Survival Forest (RSF) Models, Statistical analysis.

In feature extraction, we applied different settings for PET and CT images to adapt to different image characteristics of these two modalities, as illustrated in Additional file 1: Fig. S1. For PET images, we firstly applied SUV normalization based on patients’ body weight and injection doze. Then, we used a fixed bin-size of 0.25 SUV in intensity discretization to reduce the effect of the image noise [46]. The common parameter for bin size [47, 48] was used to ensure the reproducibility of our model. On the other hand, for CT images, we firstly shifted + 1000 HU on image values to prevent the pixel value from being negative when squared, as the minimum value of HU was -1000. For CT image discretization, we used a fixed bin size of 25 HU, as suggested in previous reports [49–51].

1246 radiomic features were extracted from ROIs delineated by clinicians on PET and CT images respectively, resulting in 2492 radiomic features per patient. Radiomic features include three major types: first-order features, shape features and texture features. First-order features describe the intensity distribution of voxels. Shape features describe the tumor shape characteristics such as volumes and surface areas. Texture features describe the second-order intensity distribution of voxels via Gray Level Co-occurrence Matrix (GLCM), Gray Level Size Zone Matrix (GLSZM), Gray Level Run Length Matrix (GLRLM), and Gray Level Dependence Matrix (GLDM). Wavelet features and Laplacian of Gaussian (LoG) features are texture features extracted from filtered images using wavelet filters and LoG filters. The radiomic feature extraction was implemented with open-source PyRadiomics library [52] (https://github.com/Radiomics/pyradiomics), which is in compliant with Imaging Biomarker Standardization Initiative [53].

### Feature selection

**Fig. 2:**
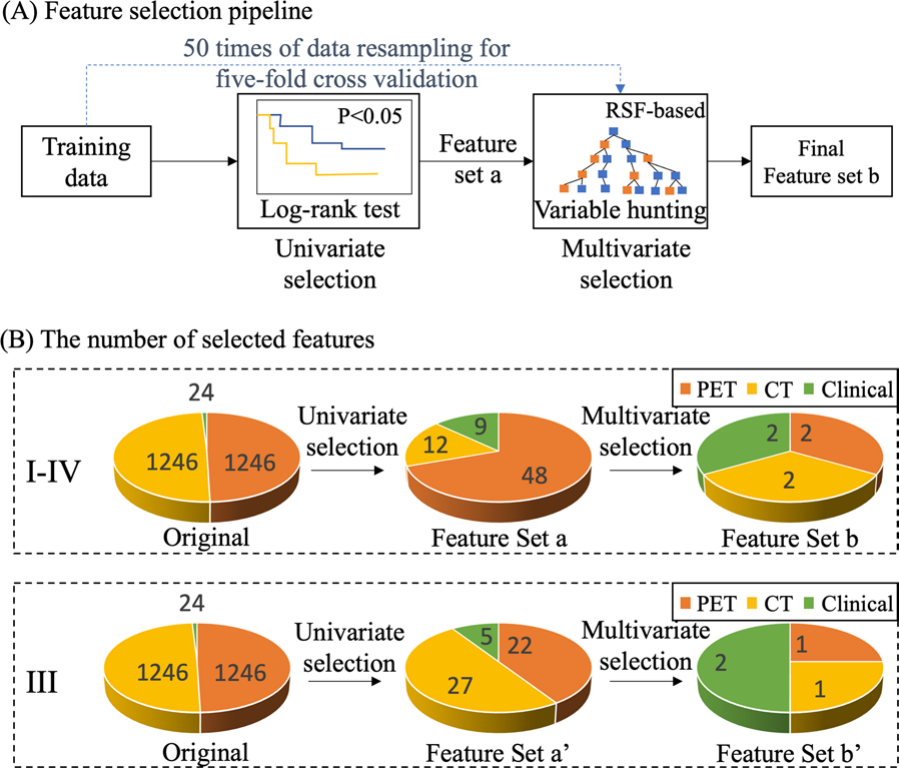
Methodology and results of feature selection. **A** Methodology of feature selection: univariate log-rank test was applied to select features with p < 0.05 to form feature set a. Multivariate variable hunting was used to select discriminative feature combination (final feature set b) via five-fold cross validation. **B** Results of feature selection: the number of selected features in feature selection pipeline

Before implementing feature selection, 24 clinico-biological features and 2492 radiomic features were fused to form a feature pool. The feature selection strategy was designed to be outcome-driven, aiming to mine features that capture the prognostic patterns. As illustrated in Fig. 2A, we applied a sequential combination of univariate and multivariate selection on the PET, CT radiomic features and clinico-biological features extracted from training data.

In univariate selection, the log-rank test was used to select statistically significant features with high prognostic values (p < 0.05). Based on the selected features, multivariate selection was deployed to select the final discriminative feature set using RSF-based variable hunting algorithm [54]. To prevent the risk of overfitting, we applied 50 times of five-fold cross-validation in multivariate feature selection to boost the generalizability of selected feature subsets. As the selected features were based on the performance of rotating training sets instead of a single fixed training set, the selected feature subset was more generalizable, thus properly avoiding the risk of overfitting.

### Modeling and validation

The patients were split into training and validation sets (7:3 ratio) using the stratified method. A random survival forest (RSF) model, which captures non-linear effects, was fitted to predict the recurrence-free survival (RFS) on the training set. To select the best performing RSF model with optimized hyperparameters, we used the grid search strategy based on the average C-index on the training set with 1000 times of bootstrap. The model performance was evaluated by C-index on the validation set with 1000 times bootstrap to reduce model overfitting. Furthermore, the predicted risks of the validation set yielded by the fitted RSF model were dichotomized into low-risk and high-risk groups. Then two groups were compared using the log-rank test to examine whether the model could stratify patients with different RFS.

### Statistical analysis

Statistical analysis was implemented using R package version 3.6.3 (R Foundation for Statistical Computing) and p-value < 0.05 was considered statistically significant. The optimal cutoff point for the log-rank test was performed by ‘surv_cutpoint’ function in the ‘survminer’ R package. The random survival forest and variable-hunting algorithm were implemented using the “randomForestSRC” R package.

## Results

### Demographics of patients

There were 196 patients with colorectal cancer involved in the dataset. Table 1 summarized the detailed demographics of patients. Of a total of 196 patients with colorectal cancer, stage I, II, III, and IV were 32 (16.3%), 44 (22.4%), 78 (39.8%), and 42 (21.4%), respectively. In the original experiment, 196 patients (ranging from stage I to stage IV) were randomly split into 138 training samples and 58 validation samples with a ratio of 7:3. The dataset used in the primary experiment is denoted as D-1 ~ 4. There were 29.6% of patients from D-1 ~ 4 who experienced recurrence. In the further experiment, we conducted the prognostic analysis on patients with stage III only, which was split into training and testing sets with a ratio of 7:3. The dataset containing only stage III patients is denoted as D-3. There were 33.3% of patients from D-3 who experienced recurrence. Compared with other studies, the patient characteristics in Table 1 listed four clinical factors including CEA, CA199, and Lymph nodes, which are automatically screened as prognostic factors by the machine program (feature selection). Our radiomics modelling combined the power of these clinical factors with imaging-based features, thus may be more valuable than clinical studies using merely clinical factors in predicting prognosis.

**Table 1:** Patients characteristics of the training and validation sets

Characteristics	Training set (n = 138)	Validation set (n = 58)	P value*
CEA	18.022 ± 41.604	25.928 ± 51.872	0.090
CA199			0.535
High	36	12	
Normal	102	46	
Lymph nodes	2.326 ± 3.560	2.172 ± 3.320	0.804
Stage			0.486
I	13	3	
II	42	23	
III	55	23	
IV	28	9	
SUVmax	14.865 ± 6.100	13.430 ± 4.772	0.229
SUVmean	8.866 ± 3.539	8.091 ± 2.831	0.270
TLG	180.122 ± 163.749	168.098 ± 153.9764	0.887

Continuous data except follow-up time (which was shown with median) were demonstrated with means ± standard deviations while categorical data were demonstrated with the number of each category and percentage. *p-value was calculated by using χ^2^ test for categorical variable and Wilcoxon test for continuous variable

### Result of feature selection

As illustrated in Fig. 2B, feature selection was applied on 2492 radiomics features extracted from PET and CT images and 24 clinico-biological features. For patients with stage I-IV, 12 CT and 48 PET radiomics features and nine clinico-biological features, were selected during univariate selection, while the final feature set composed of two CT, two PET and four clinical features was selected in multivariate selection for model building. For patients with stage III, 27 CT and 22 PET radiomics features and five clinico-biological features were selected during univariate selection, while one CT, one PET and two clinical features were selected in multivariate selection.

### Performance of radiomics signature

**Fig. 3:**
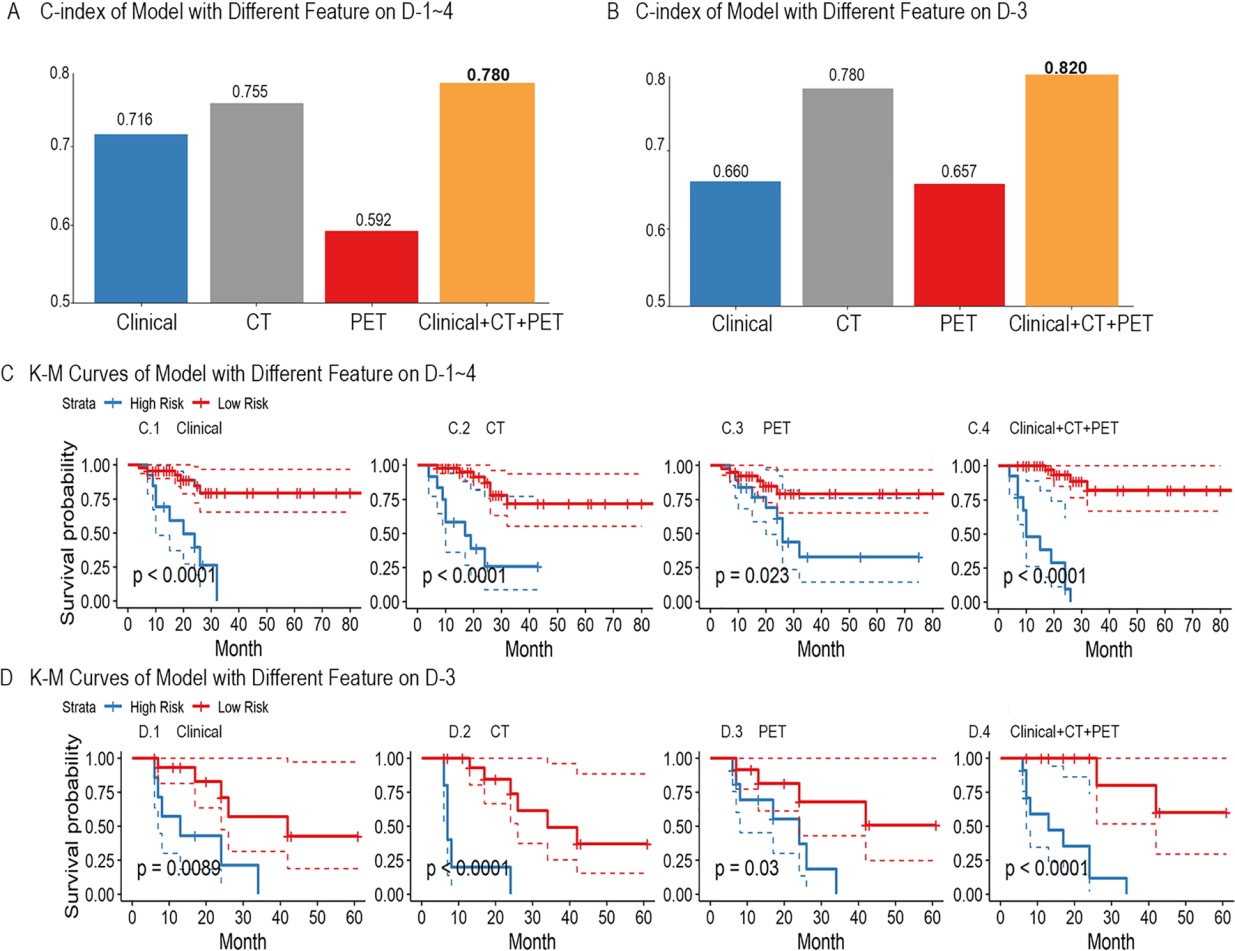
The performance of prognostic models. Figure **A** and **B** showed the comparison of the prognostic performance of different modalities on D-1 ~ 4 and D-3 respectively. Figure **C** and **D** were K–M Curves for different modalities on D-1 ~ 4 and D-3 respectively. Log-rank test was used for statistical tests used in K–M curve. P-value < 0.05 indicated survival distributions of high-risk and low-risk groups were significantly different. P-value was marked in each sub-figure of Figure **C** and **D**

The performance of selected radiomic signatures was illustrated in Fig. 3A and B for primary experiment D-1 ~ 4 and secondary experiment D3, respectively. Figure 3A and B showed RSF models built with clinical, CT and PET features outperforms models with solely clinical, PET or CT features, peaking at C-index 0.780 [95% CI 0.634–0.877] and 0.820 [95% CI 0.676–0.900] respectively. The detailed performance of signatures was attached in the Additional file 1: Table S2. K–M curves of radiomics signatures for D-1 ~ 4 and D3 were demonstrated in Fig. 3C and D respectively (*P* < 0.0001). To evaluate the risk of overfitting, we summarized training and testing C-index during the independent validation in Additional file 1: Table S1. The table showed the differences between training and testing C-index were less than 0.03 in both experiments, which suggested the risk of overfitting was properly alleviated.

### Feature analysis and interpretation

There were eight features identified by the feature selection process from D-1 ~ 4 including four clinical features (CA199, lymph nodes, stage, and Carcinoembryonic antigen (CEA)), two PET features (PET-wavelet-LLH-gldm-DV and PET-wavelet-LLL-glcm-imc2) and two CT features (CT-Log-sigma-5.0-3D-glszm-SAE, CT-Log-sigma-4.0-3D-glszm-SALGLE). The RSF model built on these eight features is denoted as M1. There were four features identified for the secondary experiment D-3 including two clinical features (CA199 and lymph nodes) and one PET feature (PET-Wavelet-LLH-glszm-ZV) and one CT feature (CT-Log-sigma-5.0-3D-glszm-SAE). The RSF model built on these four features for the secondary experiment is denoted as M2. Detailed feature explanation was attached in the Additional file 1: Table S3.

**Fig. 4:**
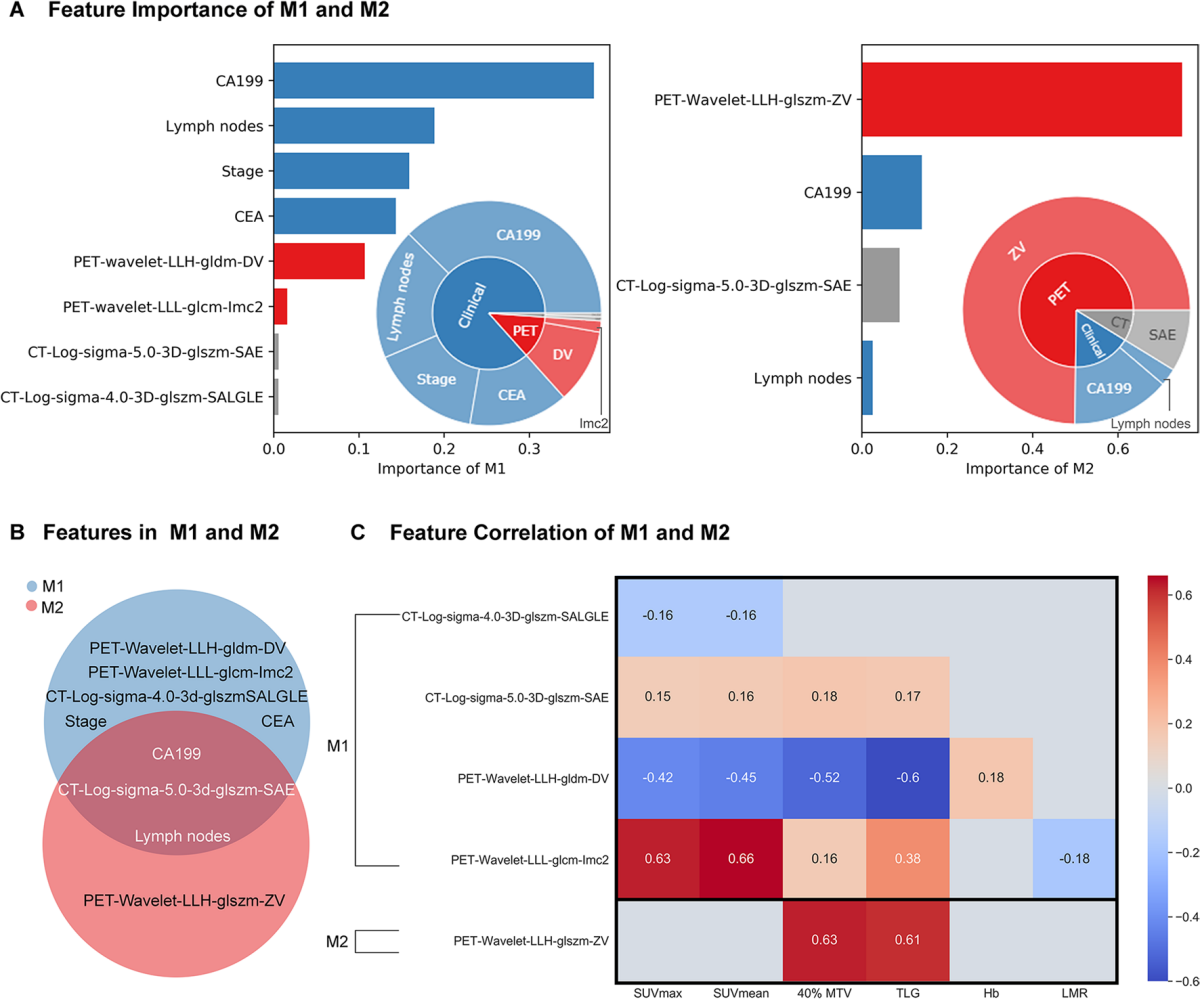
Feature importance and Pearson correlation of M1 and M2. Figure **A** was the univariate importance of features in models M1 and M2. Clinical, PET, and CT features were represented by using blue, gray, and red bar, respectively. Figure **B** showed the overlapping between features selected for models M1 and M2. In Figure **C**, Pearson test was used for correlational statistical analysis. P-value < 0.05 indicated significant correlation identified between two variables (indicated with colored cells)

We further revealed the contribution of each feature for model M1 and M2 in Fig. 4A and B. Bar graphs in Fig. 4A and B showed the normalized importance of each feature, in which CA199 contributed most in M1 and PET-Wavelet-LLH-glszm-ZV contributed most in M2. Pie graphs in Fig. 4A and B illustrated the percentage of contribution of PET, CT and clinical features. PET and CT features contributed 13.3% in M1 while contributed 83.5% in M2. In addition to feature contribution, we compared the features in M1 and M2 then found two common clinical features (CA199 and lymph nodes) and one common CT feature (CT-Log-sigma-5.0-3D-glszm-SAE).

Figure 4C summarized Pearson correlation between selected radiomic features and clinical features. It shows that radiomic features were significantly correlated to metabolic tumor activity features such as SUVmean, SUVmax, TLG, and 40%MTV.

### Case study

**Fig. 5:**
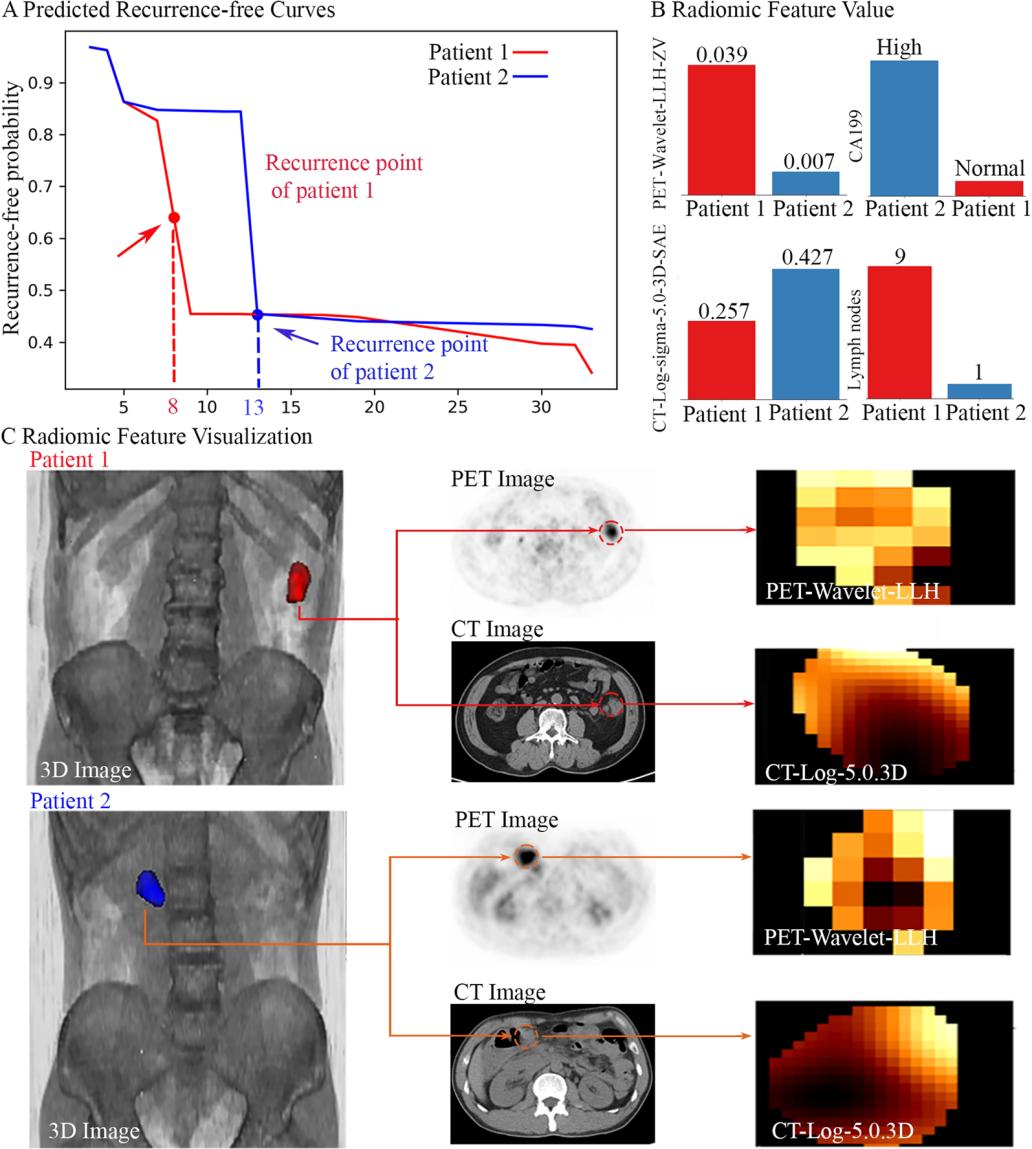
Case study for individualized result interpretation. Figure **A** showed the predicted survival curves of individual patients yielded by the model M2. Figure **B** showed the values of radiomic features of patients in the case study. Figure C visualized the tumor region in 3D body imaging, in PET/CT imaging and PET/CT radiomics features

We chose two stage IIIA samples from the validation set of the Data-3 to showcase the predictive performance of M2 model built on the radiomics signature. Our prognostic endpoint was recurrence-free survival (RFS), which focuses on the length of time before the disease recurs. As shown in Fig. 5A, our radiomics model predicted an overall lower curve in patient 1 compared patient 2. It indicated the higher chance that the disease recurred in patient 1 in a shorter time. This prediction was in accordance with the fact that real recurrence time for patient 1 (8 months) was shorter compared with patient 2 (13 months). After 15 months, both patients had low recurrence-free probability (< 50%), while patient 1 showed a trend of lower recurrence-free probability. This was also in accordance with the fact that the disease of both patients recurred, while the recurrence in patient 1 was earlier.

Values of radiomic features for these two patients were shown in Fig. 5B. The Zone variance (ZV) of PET-Wavelet-LLH image measures the variance in gray level zone size. The larger ZV, the greater heterogeneity. The SAE (Small Area Emphasis) of CT-Log-5.0-3D image measures the distribution of small size gray level zones and the larger value indicated finer textures. Detailed clinical information of two patients was included in the Additional file 1: Table S4. The visualization of radiomic features was demonstrated in Fig. 5C.

In clinic routine, patients with high CA199 tend to have worse prognosis than patients with normal CA199. In our case study, we intentionally chose two unconventional cases where patient 2 (with high CA199) had better prognosis than patient 1 (with normal CA199). For these two special cases, our PET/CT radiomics model M2, which was specifically designed to predict RFS of stage III colorectal cancer, achieved correct prediction. This is mainly because M2 combined the predictive power of both clinical and PET/CT imaging features. More specifically, as shown in Fig. 4A, among these compound features, a majority of the contribution (83.5%) from the collective PET/CT radiomics features was towards the correct prognostic decision (shorter recurrence time), while the importance of CA199 in the prediction only accounted for 14%, and thereby, the machine learning model M2 made the correct prognostic prediction.

## Discussion

Radiomics is a high-throughput mining of quantitative image features from standard medical imaging that can extract data and apply it to clinical decision support systems to improve the accuracy of diagnosis, prognosis and prediction. Radiomics is increasingly important in cancer research. Radiomics analysis leverages sophisticated image analysis tools and the rapid development and validation of medical imaging data that use image-based signatures for accurate diagnosis and treatment, providing a powerful tool for modern medicine [28–31]. Previous studies have shown that ^18^F-FDG PET/CT radiomics performed well in predicting the prognosis of various malignancies. The newly developed PET/CT radiomic signature was a powerful predictor of gastric cancer survival [32]. Radiomics features of baseline PET/CT images provide complementary prognostic information for nasopharyngeal carcinoma compared with the use of clinical parameters alone [33]. This method was also advantageous to predict the prognosis of lung cancer, breast cancer and other tumors [34–36]. As for colorectal cancer, a few studies demonstrated that FDG PET radiomic held potential towards the improved prediction of clinical outcome in stage IV patients of colorectal cancer and locally-advanced rectal cancer [42, 55]. The explosive researches on the prognostic value of PET/CT-based radiomics methods for the total colorectal cancer were rare, especially for stage III. A study on the National Cancer Data Base (NCDB) showed that CRC patients with stage III accounted for approximately one-third of all stages [56]. Moreover, the 5-year survival rate of this largest proportion of patients was subjected to a large difference in the survival outcomes [57, 58]. Therefore, it is necessary to evaluate the prognosis of stage III colorectal cancer separately, and to intervene as early as possible according to different individual patients to reduce the risk of recurrence and metastasis. In this study, we developed an original model to predict the prognosis of CRC patients and further experimented on stage III patients by ^18^F-FDG PET/CT radiomics.

We unprecedentedly investigated prognosis models for patients ranging from stage I to IV in the primary experiment. The model M1 trained by a combination of features of all three modalities outperformed other models with a C-index of 0.780 [95% CI 0.634–0.877]. The K–M curves indicated that our model effectively separated high-risk and low-risk patient groups (*P* < 0.0001). For model M1, CA199 was the most important feature. It means that this cancer antigen marker CA199 contributed most to the outcome of the prognostic prediction in model M1. The result is consistent with previous studies [43] that CA199 is a key prognostic biomarker. Notably, the contribution of imaging features was irreplaceable, although they only accounted for 13.3% of the contribution. Both PET and CT features were important and irreplicable in radiomics analysis because they both had positive importance scores, which suggests these features positively contributed to the model accuracy. Experimental results in Manuscript Fig. 3A verified that the model constructed with multimodalities (C-index 0.780) outperformed the models built with PET (C-index 0.592) or CT (C-index 0.755) alone on D-1 ~ 4. Similar trend can be identified on D-3 with Fig. 3B.

We also focused on analyzing models for patients with stage III, because the 5-year survival rate was unsatisfactory, though radical surgery and adjuvant chemotherapy were routinely performed. The prediction of prognosis is valuable for supporting individualized treatment. The C-index of M2 was 0.820 [95% CI 0.676–0.900], which means it holds a great potential value of prognostic prediction in colorectal cancer. Its performance was also superior to that of single-modality or double-modality models. K–M curves of M2 illustrated that the model could significantly separate high-risk and low-risk patient group. For model M2, PET-Wavelet-LLH-glszm-ZV was the most important feature in the predictive model, which means the texture information quantified by this PET feature successfully captured the heterogeneity of colorectal tumour towards accurate prognostic prediction. This was because PET images could provide information not only about the metabolism of the tumor, but also about the total load of the tumor. For further interpretation of this PET feature, we conducted correlation analysis, and found that this PET features positively correlated with 40% MTV and TLG (p < 0.05). CA199, which contributed most in M1, only made up 14% of all feature contributions.

Moreover, CA199, lymph nodes and CT-Log-sigma-5.0-3D-glszm-SAE were three features identified both in M1 and M2. The feature importance analysis showed that clinical features played the most vital roles in the prognosis of CRC patients of all stages, while radiomics features contributed more when predicting the prognosis of CRC patients with stage III. The case study also demonstrated that features with greater contribution could help the model to overcome the negative impact caused by single features, and then rectify the prediction. Thereby, it is reasonable to believe that the combination of clinical characteristics and imaging characteristics of ^18^F-FDG metabolism is more predictive than any single modality model.

We reduced the risk of overfitting through reducing the number of features and employed cross-validation in feature selection. Firstly, we reduced the risk of overfitting by strictly controlling the number of features, as the reduced number of features leaded to the decrease of the number of required parameters inside machine learning models, thus minimizing the risk of overfitting [59]. According to the guideline for radiomics studies [60], we reduced the number of features to less than 1/10 of sample sizes. Secondly, 50 times five-fold cross-validation was deployed during the feature selection on the training dataset to reduce the risk of overfitting [59]. By selecting features on the rotating training instead of a fixed training set, we effectively minimize the risk of overfitting on a fixed proportion of data. Thirdly, we evaluated the risk of overfitting by comparing the performance of the model on training and testing datasets in independent validation. Additional file 1: Table S1 showed that the difference between training and testing C-index was less than 0.03 in both experiments, which suggests that the risk of overfitting was properly handled.

This study was partly limited by its retrospective design and relatively modest sample sizes. We will continue to collect more patients who meet the criteria, and will attempt to conduct prospective studies to further validate our models and investigate the prognosis of patients with sub-stages (e.g., IIIA, IIIB, IIIC). We look forward to conducting further randomized controlled trials in the future on the significance and importance of ^18^F-FDG PET/CT imaging omics in the diagnosis and treatment of colorectal cancer. We will investigate the effect of spatial resolution of PET/CT images on the parameters of radiomic feature extraction.

## Conclusion

Radiomics-based decision supporting system is a powerful tool in modern medicine to identify new imaging biomarkers for more effective, accurate, and efficient diagnosis and prognostic prediction. Our developed recurrence-free survival model demonstrates that ^18^F-FDG PET/CT radiomics combined with clinical features in the study may fuel the identification of new imaging biomarkers, and could be instructive in the predictive prognosis of colorectal cancer, especially in stage III. The power of combining ^18^F-FDG PET/CT radiomics and modeling could potentially optimize the individual treatment strategies by avoiding ineffective or excessive management.

## Supplementary Information

**Additional file 1.** Additional figures and tables.

## Data Availability

The datasets generated and/or analysed during the current study are not publicly available due patient privacy and copyright issues but are available from the corresponding author on reasonable request.

## Abbreviations

CEA: Carcinoembryonic antigen
CRC: Colorectal Cancer
^18^F-FDG: ^18^F-fluoro-2-deoxy-d-glucose
PET/CT: Positron emission tomography/computed tomography
SUV: Standardized uptake value
MTV: Metabolic tumor volume
TLG: Total lesion glycolysis
VOI: The volume of interest
RSF: Random survival forest
