# Supplementary material for: Radiomic analysis for predicting prognosis of colorectal cancer from preoperative 18F-FDG PET/CT
Source: J Transl Med. 2022 Feb 2;20:66. doi: 10.1186/s12967-022-03262-5 (PMC8812058; doi:10.1186/s12967-022-03262-5)
Supplement: Supplementary file 1 — Additional file 1. Additional figures and tables. [file 12967_2022_3262_MOESM1_ESM.docx]

**Supplementary material**

**Fig. S1** Different settings for feature extraction of PET and CT images.

**Table S1.** Training and testing C-index for overfitting analysis**.**

|  | Training C-index | Testing C-index | C-index Difference |
| --- | --- | --- | --- |
| I-IV | 0.792 | 0.780 | **0.012** |
| III | 0.848 | 0.820 | **0.028** |

**Table S2** Model performance with different feature type

| **D-1~4** | **Clinical** | **CT** | **PET** | **Mixed**  **(Clinical + Radiomics)** |
| --- | --- | --- | --- | --- |
| C-index | 0.716 | 0.755 | 0.592 | **0.780** |
| 95% CI | 0.565,0.841 | 0.588,0.883 | 0.375,0.733 | **0.634,0.877** |
| **D-3** |  |  |  |  |
| C-index | 0.660 | 0.78 | 0.657 | **0.820** |
| 95% CI | 0.325,0.862 | 0.588, 0.913 | 0.344,0.841 | **0.676,0.900** |

**Table S3** Meaning of selected radiomic features

| **Radiomic Feature** | **Model** | **Formula** | **Feature meaning** |
| --- | --- | --- | --- |
| Log-sigma-5.0-3D-glszm-SAE | M1, M2 | $SAE=\frac{\sum_{i=1}^{N_{g}} \sum_{j=1}^{N_{s}} P(i,j)j^{2}}{N_{z}}$ | Small Area Emphasis (SAE) is a measure of the distribution of small size zones, with a greater value indicative of more smaller size zones and more fine textures. |
| Log-sigma-4.0-3D-glszm-SALGLE | M1 | $SALGLE=\frac{\sum_{i=1}^{N_{g}} \sum_{j=1}^{N_{s}} \frac{p(i,j)}{{i^{2}j}^{2}}}{N_{z}}$ | Small Area Low Gray Level Emphasis (SALGLE) measures the proportion in the image of the joint distribution of smaller size zones with lower gray-level values. |
| Wavelet-LLH-gldm-DV | M1 | $DV=\sum_{i=1}^{N_{g}} \sum_{j=1}^{N_{d}} p\left( i,j \right)\left( j-\mu\right)^{2}$  $,where \mu=\sum_{i=1}^{N_{g}} \sum_{j=1}^{N_{d}} jp(i,j)$ | Dependence Variance (DV) measures the variance in dependence size in the image. |
| Wavelet-LLL-glcm-Imc2 | M1 | $IMC2=\sqrt{1-e^{-2(HXY2-HXY)}}$ | Informational Measure of Correlation (IMC) 2 quantifies the complexity of the texture. |
| Wavelet-LLH-glszm-ZV | M2 | $ZV=\sum_{i=1}^{N_{g}} \sum_{j=1}^{N_{s}} p\left( i,j \right)\left( j-\mu\right)^{2}$  $, where \mu=\sum_{i=1}^{N_{g}} \sum_{j=1}^{N_{s}} jp(i,j)$ | Zone Variance (ZV) measures the variance in zone size volumes for the zones. |

**Table S4** Information of patients in case study

| **ID** | Patient 1 | Patient 2 |
| --- | --- | --- |
| **follow-up time** | 8 | 13 |
| **CA199** | normal | high |
| **lymph nodes** | 9 | 1 |
| **stage NO** | 3 | 3 |
| **40%MTV** | 12.28 | 10 |
| **SUVmax** | 6.41 | 6.47 |
| **SUVmean** | 3.95 | 3.77 |
| **TLG** | 48.506 | 37.7 |
| **recurrence** | 1 | 1 |
| **gender** | 1 | 1 |
| **age** | 74 | 43 |
| **Hb** | 153 | 155 |
| **PLT** | 274 | 269 |
| **NLR** | 1.652173913 | 24.5 |
| **PLR** | 119.1304348 | 448.3333333 |
| **LMR** | 7.666666667 | 1.5 |
| **CA50** | normal | high |
| **CEA** | 5.55 | 22.97 |
| **location** | sigmoid | transverse |
